# Effect of* Laminaria digitata* dietary inclusion and CAZyme supplementation on blood cells, serum metabolites and hepatic lipids and minerals of weaned piglets

**DOI:** 10.1038/s41598-023-33835-3

**Published:** 2023-04-22

**Authors:** David M. Ribeiro, Rui M. A. Pinto, Paula A. Lopes, José M. Pestana, Cristina M. Alfaia, Mónica M. Costa, Daniela F. P. Carvalho, Miguel P. Mourato, André M. de Almeida, João P. B. Freire, José A. M. Prates

**Affiliations:** 1 LEAF-Linking Landscape, Environment, Agriculture and Food Research Center, Associated Laboratory TERRA, Instituto Superior de Agronomia, Universidade de Lisboa, Tapada da Ajuda, 1349-017 Lisboa, Portugal; 2 iMED.UL, Faculdade de Farmácia, Universidade de Lisboa, Avenida Professor Gama Pinto, 1649-003 Lisboa, Portugal; 3 JCS, Laboratório de Análises Clínicas Dr. Joaquim Chaves, Avenida General Norton de Matos, Miraflores, 1495-148 Algés, Portugal; 4 CIISA-Centre for Interdisciplinary Research in Animal Health, Faculdade de Medicina Veterinária, Universidade de Lisboa, 1300-477 Lisboa, Portugal; 5 Laboratório Associado para Ciência Animal e Veterinária (AL4AnimalS), Faculdade de Medicina Veterinária, Universidade de Lisboa, Av. da Universidade Técnica, 1300-477 Lisboa, Portugal

**Keywords:** Animal physiology, Metabolism

## Abstract

Seaweeds, such as *Laminaria digitata*, are a sustainable alternative to conventional feedstuffs for weaned piglet diets, improving their health and mitigating environmental impacts. *L. digitata* has a complex cell wall that can be difficult for monogastrics to digest. However, carbohydrate-active enzymes (CAZymes) such as Rovabio^®^ Excel AP and alginate lyase can help break down these polysaccharides and render intracellular nutrients more accessible. This study aimed to evaluate the impact of 10% *L. digitata* feed inclusion and CAZyme supplementation on piglet blood cells, serum metabolites, liver lipid and mineral profiles. Forty weaned piglets were randomly assigned to one of four diets (n = 10 each): a control diet, 10% *L. digitata* (LA), 10% *L. digitata* + 0.005% Rovabio^®^ Excel AP (LAR), and 10% *L. digitata* + 0.01% alginate lyase (LAL). After two weeks of trial, animals were slaughtered and liver and blood serum samples taken for analysis. The results showed that the LA and LAL diets increased blood lymphocytes, IgG and IgM, and decreased serum lipids, improving both cellular and humoral immune response and cardiovascular health. Dietary CAZymes reversed the anti-inflammatory and hematopoietic effects. Additionally, cortisol levels were reduced with seaweed inclusion compared to the control diet (P < 0.001). In the liver, total n-3 PUFA and n-6/n-3 ratio were increased and decreased, respectively, due to eicosapentaenoic acid and α-linolenic acid accumulation (P < 0.001). However, total liver mineral content was incorporated to a lesser extent with the combined seaweed and enzyme diets (P < 0.001), potentially indicating a negative effect on mineral bioavailability. Overall, results suggest that a 10% *L. digitata* inclusion can effectively improve piglet health by reducing stress during weaning, without the need for dietary CAZymes.

## Introduction

In recent years, there has been a growing concern about the environmental sustainability of livestock production, particularly regarding alternatives to conventional feedstuffs like corn or soybean meal. These are imported at high costs, both financially and environmentally, from outside of Europe. Considering that feeding is the main cost in pig production^1^ and the projected increase in pork demand, establishing cost-effective and locally produced feedstuffs is of utmost importance, especially in the context of a circular economy^2,3^.

Seaweeds are a heterogenous group of organisms that provide an abundant source of biomass. They can be classified in three major groups: *Phaeophyceae* (brown algae), *Rhodophyceae* (red algae), and *Chlorophyceae* (green algae). They have a recalcitrant cell wall that limits nutrient availability during digestion by monogastric animals^4^. Carbohydrate-active enzymes (CAZymes) are a putative solution to this problem, if effective in the in vivo disruption of the cell wall, via supplementation in the feed^5^. Seaweeds such as *Laminaria digitata* are novel feed ingredients, with the potential to improve pig meat quality and health status, by decreasing lipid oxidation and improving gut health, similarly to what has been reported for microalgae^6,7^. Indeed, macroalgae have bioactive compounds with health promoting properties, namely polysaccharides, minerals, pigments and n-3 polyunsaturated fatty acids (PUFA)^4^. For additional details, please refer to our research team’s review on this subject^4^. This is particularly important in the context of post-weaning piglets, given that during this phase, animals endure several challenges, including social, environmental and nutritional changes^8^. Our research team has recently reported the effects of a single alginate lyase in vitro, towards the partial degradation of *L. digitata* cell wall, promoting the release of monounsaturated fatty acids (MUFA)^9^.

Recent research has shown that supplementing sow diets with polysaccharide extracts from *Laminaria* sp. increases serum IgG and IgA in suckling piglets^10,11^. Other studies have shown that dietary *Laminaria japonica* increases nitric oxide and has no effect on piglet serum urea concentrations^12,13^. Our research team has recently reported the effects of dietary microalgae (*Chlorella vulgaris* and *Arthrospira platensis*) and CAZyme supplementation on serum and hepatic biochemical profiles of piglets^14,15^. The most important findings were an immunosuppressive effect caused by 5% *C. vulgaris*, by lowering the serum concentrations of immunoglobulins (IgA, IgG and IgM). In turn, it also increased the accumulation of n-3 PUFA (including eicosapentaenoic acid, EPA) and reduced hepatic n-6/n-3 ratio^14^. On the other hand, dietary *A. platensis* increased serum total lipids, total cholesterol and LDL-cholesterol of weaned piglets, whilst improving antioxidant potential^15^. Even though *L. digitata* has a very different composition, analogous or additional effects can also be found.

As already mentioned, we have previously reported the efficiency of a recombinant alginate lyase in degrading the cell wall in vitro^9^. Furthermore, a recent study by our research team showed that a 10% *L. digitata* feed inclusion and CAZyme supplementation led to high deposition of n-3 PUFA and minerals in pork^16^. Given that seaweeds have a well described bioactive composition^2^, with even immune modulatory traits, we hypothesized that a diet with 10% *L. digitata* (LA) could have beneficial effects on weaned piglet systemic and hepatic metabolism, and that these could be further improved by the supplementation of either the recombinant alginate lyase (LAL) or a commercially available CAZyme mixture (Rovabio^®^ Excel AP, LAR). Therefore, this study aimed to evaluate the effect of this supplementation on blood cells, detailed serum metabolites and hepatic metabolism by quantifying lipid and mineral profiles of recently weaned piglets.

## Results

### Blood cells and serum metabolite profile

The effects of *L. digitata* dietary inclusion with or without CAZyme supplementation on blood cells and serum metabolites from piglets are shown in Table 1. The number of white blood cells (P = 0.003) and red blood cells (P = 0.009) was higher in piglets fed with seaweed without enzyme supplementation when compared to the control group. Thrombocytes were also significantly higher in this group compared to both control and alginate lyase supplemented groups (P = 0.006). The percentage of monocytes was not significantly different between groups (P > 0.05). In turn, granulocytes were reduced in piglets fed with *L. digitata* inclusion and in the group supplemented with alginate lyase when compared to the control group (P < 0.001). The opposite was observed for the percentage of lymphocytes (P < 0.001), where feeding seaweed alone or in combination with the recombinant lyase resulted in significantly higher values than the control group. Haemoglobin followed the same trend of red blood cells and was increased by *L. digitata* inclusion (without enzyme supplementation) in relation to the combination of *Laminaria* with recombinant lyase (P = 0.007).

**Table 1:** Effect of dietary treatments on haematologic and serologic profiles of pigs. *Ctrl* control diet, *LA* control + 10% *Laminaria digitata*, *LAR* LA + 0.005% Rovabio^®^ Excel AP, *LAL* LA + 0.01% alginate lyase, *TAG* triacylglycerols, *HDL* high-density lipoprotein, *LDL* low density lipoprotein, *VLDL* very low density lipoprotein, *ALT* alanine aminotransferase, *ALP* alkaline phosphatase, *AST* aspartate aminotransferase, *GGT* gamma-glutamyltransferase, *IGF-1* insulin growth factor 1, *IL-10* interleukin 10, *IL-6* interleukin 6, *IgG* immunoglobulin G, *IgM* immunoglobulin M, *ApoA1* apolipoprotein A1, *SEM* standard error of the means. Values with different superscripts are significantly different at P < 0.05.

	Ctrl	LA	LAR	LAL	SEM	P value
Haematology						
White blood cells (× 10^9^/L)	15.4^b^	20.2^a^	18.4ª^b^	17.1^ab^	0.903	0.003
Granulocytes (%)	48.7^a^	38.7^c^	46.8^ab^	42.8^bc^	1.41	< 0.001
Lymphocytes (%)	46.5^c^	57.4^a^	50.0^bc^	53.4^ab^	1.59	< 0.001
Monocytes (%)	4.50	4.00	2.80	3.60	0.764	0.432
Red blood cells (× 10^12^/L)	6.11^b^	7.31^a^	6.97^ab^	6.31^ab^	0.274	0.009
Haemoglobin (g/L)	112^ab^	122^a^	115^ab^	106^b^	0.320	0.007
Thrombocytes (× 10^9^/L)	304^b^	401^a^	352^ab^	300^b^	22.5	0.006
Serum metabolites						
Glucose (mg/dL)	126^a^	112^b^	125^a^	125^a^	2.52	< 0.001
Insulin (µU/mL)	0.480	0.517	0.603	0.400	0.066	0.199
HOMA-IR^A^ (mmol/L × µU/mL)	0.148	0.144	0.192	0.125	0.023	0.236
Urea (mg/dL)	7.10	6.30	7.40	5.80	0.514	0.127
Creatinine (mg/dL)	0.780^a^	0.594^c^	0.680^b^	0.561^c^	0.019	< 0.001
Cholesterol (mg/dL)	67.8^a^	61.6^b^	70.8^a^	62.1^b^	2.42	0.027
LDL-cholesterol (mg/dL)	34.1^ab^	32.1^ab^	36.0^a^	29.7^b^	1.30	0.010
HDL-cholesterol (mg/dL)	28.4	25.3	28.9	28.7	1.24	0.150
VLDL-cholesterol^B^ (mg/dL)	10.9^a^	7.62^c^	8.84^bc^	10.8^ab^	0.559	< 0.001
Total lipids^C^ (mg/dL)	341^a^	308^b^	336^a^	328^ab^	6.33	0.004
TAG (mg/dL)	54.9^a^	38.1^c^	44.2^bc^	54.1^ab^	2.79	< 0.001
Albumin (g/dL)	3.16	3.22	3.13	2.99	0.077	0.190
Total protein (g/dL)	4.83^a^	5.09^a^	4.17^b^	4.29^b^	0.116	< 0.001
Hepatic markers						
ALT (U/L)	23.3^a^	10.7^b^	20.4^a^	14.4^b^	1.20	< 0.001
AST (U/L)	57.0^ab^	36.7^b^	70.2^a^	51.6^ab^	5.57	0.002
GGT (U/L)	31.6^a^	16.9^b^	27.6^a^	29.2^a^	1.83	< 0.001
ALP (U/L)	267^b^	206^c^	339^a^	284^b^	9.85	< 0.001
Immunoglobulins						
IgA (mg/dL)	< 4.0	< 4.0	< 4.0	< 4.0	–	–
IgG (mg/dL)	148^b^	191^a^	78.2^c^	124^b^	11.3	< 0.001
IgM (mg/dL)	27.4^b^	41.6^a^	35.0^ab^	39.2^a^	2.36	< 0.001
Hormones and inflammation markers						
Cortisol (µg/dL)	3.76^a^	1.01^b^	1.56^b^	1.63^b^	0.286	< 0.001
IGF-1 (µg/L)	139^b^	181^a^	149^ab^	160^ab^	10.3	0.040
IL-10 (pg/mL)	17.1^b^	25.6^a^	25.3^a^	20.6^ab^	1.63	0.002
C-reactive protein (mg/dL)	< 0.03	< 0.03	< 0.03	< 0.03	–	–
IL-6 (pg/mL)	< 1.5	< 1.5	< 1.5	< 1.5	–	–
ApoA1 (mg/dL)	< 3.0	< 3.0	< 3.0	< 3.0	–	–
Electrolytes						
Na^+^ (mEq/L)	144^a^	142^b^	143^ab^	142^b^	0.653	0.030
K^+^ (mEq/L)	6.81^b^	6.98^ab^	7.63^a^	7.07^ab^	0.196	0.032
Cl^−^ (mEq/L)	101^a^	99.5^ab^	101^a^	97.4^b^	0.725	0.001
Redox markers						
TAC (µM)	145	147	143	141	4.54	0.774
GPx (U/L)	268	326	338	302	71.8	0.903

^A^HOMA-IR, insulin resistance index = [fasting serum glucose] × [fasting serum insulin]/22.5.

^B^VLDL-cholesterol = 1/5 (TAG).

^C^Total lipids = (total cholesterol) × 1.12 + (TAG) × 1.33 + 1.48.

Insulin was not affected by dietary treatments (P > 0.05). In turn, glucose levels were found to be reduced by *L. digitata* (P < 0.001) but was reversed with Rovabio^®^ or recombinant lyase supplemented diets, which obtained similar levels to those of the control group. Even though urea concentrations did not change (P > 0.05), creatinine was reduced in *L. digitata* fed groups compared to controls (P < 0.001). Regarding lipemia, dietary *L. digitata* without enzyme supplementation consistently reduced lipid parameters, such as total cholesterol, LDL-cholesterol, VLDL-cholesterol, total lipids and TAG, in comparison to the control group (at least P = 0.027). Albumin was unchanged across dietary groups (P > 0.05), whereas total protein (P < 0.001) decreased with both commercial Rovabio^®^ and recombinant lyase supplementation. Hepatic markers, such as ALT (P < 0.001), AST (P = 0.002), GGT (P < 0.001) and ALP (P < 0.001) were all significantly decreased by seaweed dietary inclusion reaching the lowest values in piglets fed without CAZymes supplementation. IgA, C-reactive protein, IL-6 and ApoA1 were found below the minimal levels of detection. The immunoglobulins, IgG (P < 0.001) and IgM (P < 0.001), reached the highest values in piglets fed with *L. digitata* without enzyme supplementation in relation to the control. IGF-1 (P = 0.040) and IL-10 (P = 0.002) presented the same difference, reaching the highest levels in piglets fed the seaweed with no enzyme inclusion, intermediate when combined with CAZymes, and lowest in the control group. The stress hormone, cortisol decreased with *L. digitata* incorporation, regardless of CAZymes inclusion (P < 0.001). Regarding the electrolytes, the values found are very similar across the different experimental groups. For Potassium (P = 0.032), the highest levels were observed in piglets fed the control diet and *L. digitata* inclusion with the commercial Rovabio^®^ in comparison to *L. digitata* inclusion and the recombinant lyase group. The assessment of redox balance did not lead to significant differences (P > 0.05) among dietary groups for total antioxidant capacity and glutathione peroxidase activity.

### Hepatic cholesterol and fatty acids

Hepatic total lipids, cholesterol and fatty acid profiles are presented in Table 2. Total lipids and cholesterol did not change across experimental groups (P > 0.05). However, there was a strong tendency for reduced total lipid contents in the LAR piglets when compared to the remaining groups (P = 0.051). The *L. digitata* diet contributed to decreased levels of medium chain saturated fatty acids, namely 12:0 (P = 0.001) and 14:0 (P < 0.001) when compared to the control group. In turn, there was a tendency for increased long-chain saturated fatty acids (SFA) 22:0 (P = 0.074) and 20:0 (P = 0.071) in the *L. digitata* without enzyme diet and the one supplemented with Rovabio^®^, respectively, when compared to the other diets. Total SFA showed no differences between experimental groups.

**Table 2:** Effect of diets on the hepatic fatty acid profile (g/100 g liver). *Ctrl* control diet, *LA* control + 10% *Laminaria digitata*, *LAR* LA + 0.005% Rovabio^®^ Excel AP, *LAL* LA + 0.01% alginate lyase. *SEM* standard error of the means. Values with different superscripts are significantly different at P < 0.05.

	Ctrl	LA	LAR	LAL	SEM	P-value
Total lipids (g/100 g)	2.16	2.10	1.93	2.06	0.059	0.051
Total cholesterol (mg/100 g)	2.29	2.19	2.22	2.13	0.089	0.631
Fatty acid composition (g/100 g FA)						
Saturated fatty acids						
12:0	0.080^a^	0.042^b^	0.060^ab^	0.045^b^	0.0094	0.001
14:0	0.23^bc^	0.19^c^	0.25^ab^	0.28^a^	0.016	< 0.001
15:0	1.04	1.00	1.12	1.27	0.189	0.487
16:0	17.3	16.6	17.5	18.1	1.00	0.517
17:0	6.28	6.74	7.48	8.17	1.014	0.276
18:0	31.7	30.4	31.1	29.3	1.63	0.508
20:0	0.082	0.081	0.113	0.065	0.0179	0.071
22:0	0.058	0.104	0.064	0.094	0.0181	0.074
Total SFA	56.7	55.2	57.7	57.4	1.87	0.550
Monounsaturated fatty acids						
16:1c7	0.46	0.37	0.42	0.36	0.046	0.157
16:1c9	0.62^b^	0.64^ab^	0.71^ab^	0.82^a^	0.066	0.025
17:1c9	0.88	1.00	1.08	1.16	0.175	0.436
18:1c9	13.2^a^	11.8^b^	12.8^ab^	12.3^ab^	0.41	0.010
18:1c11	2.30	2.03	2.18	2.09	0.134	0.218
20:1c11	0.12	0.12	0.12	0.10	0.027	0.820
22:1n-9	0.025	0.055	0.051	0.056	0.0153	0.150
Total MUFA	17.6	16.0	17.3	16.9	0.59	0.054
Polyunsaturated fatty acids						
18:2n-6	14.0	14.3	13.1	13.3	0.90	0.540
18:3n-6	0.068	0.060	0.081	0.064	0.0131	0.393
18:2t9t12	0.10	0.10	0.13	0.11	0.019	0.306
18:3n-3	0.18^b^	0.35^ab^	0.43^a^	0.40^a^	0.066	0.003
18:4n-3	0.041	0.065	0.084	0.065	0.0164	0.090
20:2n-6	0.78	0.67	0.65	0.67	0.122	0.730
20:3n-6	0.32	0.37	0.35	0.32	0.054	0.703
20:4n-6	7.27	9.52	7.08	7.66	1.123	0.134
20:3n-3	0.009	0.073	0.050	0.046	0.0243	0.086
20:5n-3	0.030^b^	0.095^a^	0.143^a^	0.115^a^	0.0240	< 0.001
22:5n-3	0.18^b^	0.23^ab^	0.27^ab^	0.32^a^	0.039	0.008
22:6n-3	0.10	0.17	0.24	0.19	0.049	0.062
Total n-3 PUFA	0.54^b^	0.98^a^	1.21^a^	1.13^a^	0.108	< 0.001
Total n-6 PUFA	22.4	24.9	21.3	22.0	2.07	0.347
Total PUFA	23.1	25.9	22.6	23.2	2.06	0.370
Other FA	2.64	2.89	2.34	2.49	0.519	0.748
Fatty acid ratios						
PUFA:SFA	0.41	0.49	0.39	0.41	0.057	0.327
n-6:n-3	48.5^a^	28.5^b^	19.2^b^	19.9^b^	6.48	< 0.001

Regarding MUFA, 16:1c9 increased by 32% in recombinant lyase fed piglets when compared to controls (P = 0.025), whereas the latter group had a 12% increase in 18:1c9 when compared to animals fed *L. digitata* with no enzyme supplementation (P = 0.010), contributing to lower total MUFA compared to controls (P = 0.054). PUFA were generally increased due to *L. digitata* inclusion, regardless of enzyme supplementation. For instance, 18:3n-3 and 20:5n-3 were respectively increased in 2.39 and 4.77-fold in piglets fed seaweed diets when compared to controls (P = 0.003 and P < 0.001, respectively). Alginate lyase supplementation significantly improved 22:5n-3 concentration when compared to the control group. All of these variations increased total n-3 PUFA (P < 0.001) and reduced n-6/n-3 ratio (P < 0.001) in seaweed-fed groups.

### Hepatic α-tocopherol and pigments

Liver pigments and α-tocopherol contents are presented in Table 3. Similar to α-tocopherol, no differences (P > 0.05) were found for liver pigments, including chlorophyll (a and b), total chlorophyll, total carotenoids and the chlorophylls plus carotenoids sum.

**Table 3:** Effect of dietary treatments on hepatic α-tocopherol (mg/100 g) and pigments (µg/100 g liver). *Ctrl* control diet, *LA* control + 10% *Laminaria digitata*, *LAR* LA + 0.005% Rovabio^®^ Excel AP, *LAL* LA + 0.01% alginate lyase, *SEM* standard error of the means. Values with different superscripts are significantly different at P < 0.05.

	Ctrl	LA	LAR	LAL	SEM	P-value
α-Tocopherol	164	168	154	151	0.095	0.565
Pigments						
Chlorophyll-a	17.8	16.3	19.7	19.1	2.48	0.764
Chlorophyll-b	43.4	46.0	53.6	43.7	7.75	0.765
Total chlorophylls	61.2	62.2	73.4	62.8	9.58	0.787
Total carotenoids	115	117	117	117	5.41	0.984
Total chlorophylls + carotenoids	176	179	191	179	11.9	0.826

### Mineral profile

Mineral profiles are presented in Table 4. Enzymatic supplementation significantly reduced hepatic Phosphorous content by 31% when compared to the control group (P < 0.001). A similar trend was found for Sulphur, with piglets fed *L. digitata* with both enzyme supplemented groups having the lowest concentrations (P = 0.039). This resulted in less total macrominerals in the liver of piglets fed with seaweed and with enzyme supplementation (Rovabio^®^ and alginate lyase) compared to either control or *L. digitata* fed groups (P < 0.001). Regarding microminerals, a 32% Copper content increase was observed in the control diet when compared to *L. digitata* supplemented with the recombinant lyase (P = 0.002). In turn, piglets fed on *L. digitata* with recombinant lyase had less 0.04 mg Manganese compared to *L. digitata* alone (P = 0.023). Following the same trend mentioned for macrominerals, animals fed *L. digitata* with both Rovabio^®^ and recombinant lyase showed a lower total micromineral concentration compared to either control or non-supplemented *L. digitata* groups (P < 0.001).

**Table 4:** Effect of diet on the hepatic mineral profile (mg/100 g of liver) of piglets. *Ctrl* control diet, *LA* control + 10% *Laminaria digitata*, *LAR* LA + 0.005% Rovabio^®^ Excel AP, *LAL* LA + 0.01% alginate lyase, *SEM* standard error of the means. Values with different superscripts are significantly different at P < 0.05.

	Ctrl	LA	LAR	LAL	SEM	P-value
Macrominerals						
Calcium (Ca)	19.1	20.1	18.3	19.1	0.81	0.512
Potassium (K)	389	392	392	396	3.6	0.412
Magnesium (Mg)	21.0	21.7	20.7	20.8	0.43	0.357
Sodium (Na)	76.6	81.9	77.7	75.1	2.00	0.118
Phosphorous (P)	354^a^	348^a^	288^b^	209^c^	11.8	< 0.001
Sulphur (S)	175^a^	184^a^	162^b^	164^b^	5.7	0.039
Total	1035^a^	1048^a^	963^b^	884^c^	16.7	< 0.001
Microminerals						
Copper (Cu)	2.61^a^	1.66^b^	2.09^ab^	1.98^b^	0.159	0.002
Iron (Fe)	11.0	11.5	9.3	10.0	0.64	0.078
Manganese (Mn)	0.33^ab^	0.34^a^	0.31^ab^	0.30^b^	0.010	0.023
Zinc (Zn)	14.6	15.3	14.6	15.5	1.32	0.946
Total	28.5	28.9	26.3	27.8	1.55	0.657
Total minerals	1063^a^	1077^a^	988^b^	912^c^	17.5	< 0.001

### Principal component analysis (PCA)

**Figure 1:**
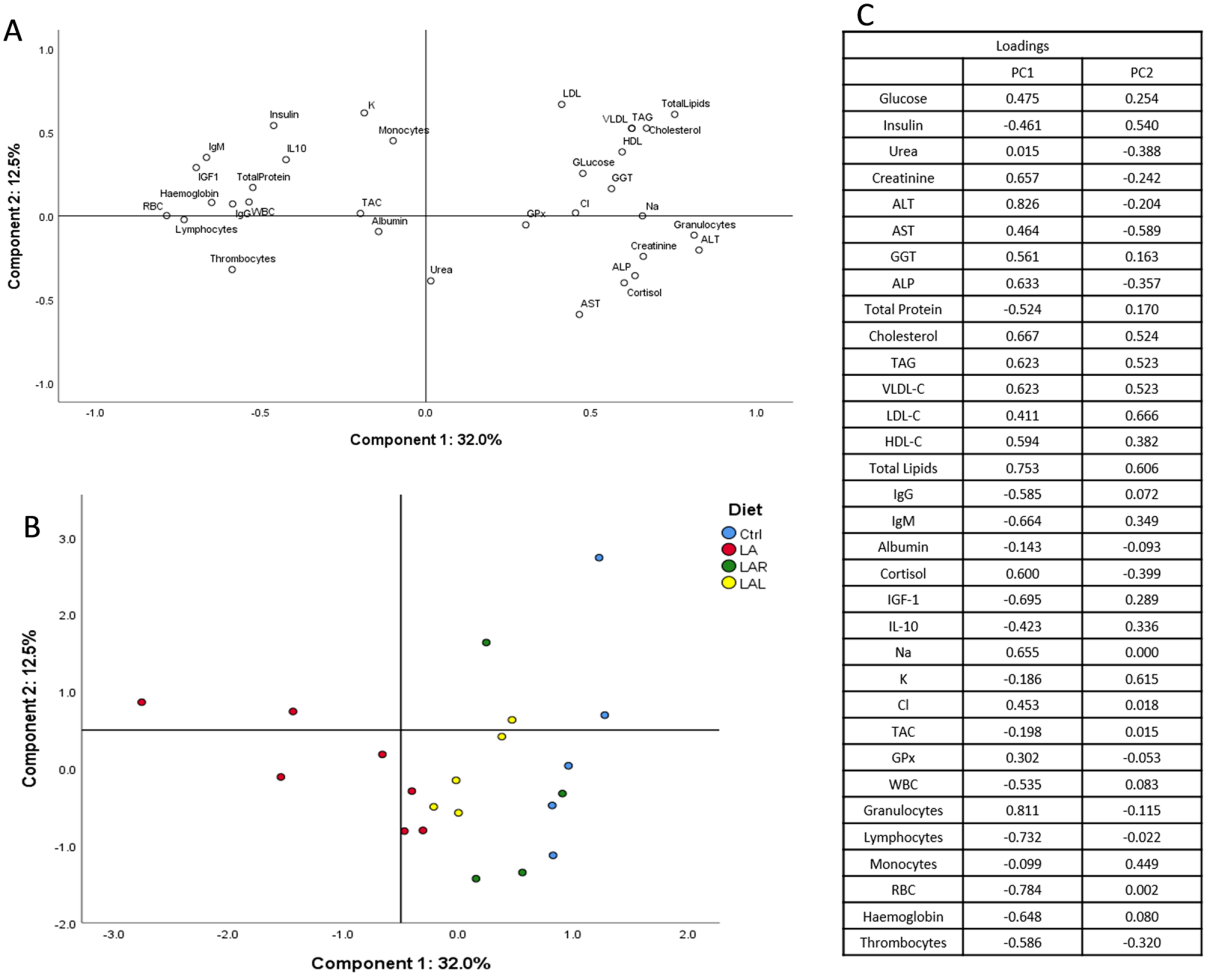
Principal component analysis (PCA) of serum profile (**A**) and distribution of experimental groups on components 1 and 2 (**B**) from piglets fed Ctrl—control diet; LA—control + 10% *Laminaria digitata*; LAR—LA + 0.005% Rovabio^®^ Excel AP; LAL—LA + 0.01% alginate lyase. Loadings are presented for components 1 and 2 (**C**).

The PCA performed with hepatic parameters showed no clear clustering of any particular group, with the first and second principal components representing 29% of data variance, reflecting its dispersion (data not shown). Conversely, the PCA performed with blood cells and serum metabolites (Fig. 1) showed a particular separation of *L. digitata* experimental group from the other groups on the first principal component, explaining 32% of data variance. This discriminant effect was caused by variables such as haemoglobin, red blood cells, IgM, IGF-1 and lymphocytes. Overall, this suggests the existence of similar serum biochemical profiles between the control and *L. digitata* supplemented with both CAZymes.

## Discussion

To the best of our knowledge, and with the exception of the previously mentioned companion paper^16^, the use of *L. digitata* as a feed ingredient with 10% of dietary incorporation in weaned piglet diets has not been previously reported. Indeed, previous studies have shown that inclusion levels below 3% have generally no effect on animal performance^17^. For instance, feeding 1.5% of a seaweed mix to piglets has yielded no major differences in growth performance^18^. In turn, feeding up to 5% *L. japonica* had no difference in growth^13^ and 10% *Ascophylum nodosum* reduced weight gain in weaned and finishing pigs^19^, respectively. In the present study, there was no effect of experimental diets in any production performance variable measured, as reported^16^.

On a continuing effort to unveil molecular mechanisms, underlying metabolic pathways associated with health and disease, experimental pigs stand out as the most valuable model in translational research, as pigs and humans share numerous anatomical and physiological similarities^20^. In this experiment, there was a strong effect of experimental diets on blood cells. Indeed, a sharp increase was detected in thrombocytes, white and red blood cells because of dietary seaweed inclusion that was counterbalanced by enzymatic supplementation. A modulatory effect of dietary seaweed on blood cell populations was previously reported by Shimazu et al.^21^. In such study, authors reported that 1% dietary wakame (*Undaria pinnatifida*) increased natural killer cells in pig blood. This originated in the immune system stimulation, either from a direct effect of seaweed components (including fucoidan and bioactive polysaccharides), and/or indirectly via microflora changes in the gut, namely by increasing the proportion of beneficial bacteria (*Lactobacillus*) and reducing detrimental ones (*Escherichia coli*). Accordingly, sow diet supplementation with seaweed extract (laminarin and fucoidan) obtained from *Laminaria* spp. has been reported to increase phagocytic activity of lymphocytes of piglets at weaning, despite reducing overall blood eosinophils^22^. According to our data, blood cell modulation can be caused by *L. digitata* polysaccharides. Indeed, LA piglets were fed without the added enzymatic mixture, whereas LAR and LAL diets contain laminarinase and alginate lyase, which degrade bioactive laminarin and alginate, respectively. By degrading these polysaccharides during digestion, these enzymes can inhibit their bioactive function that was thus kept in the LA group. This may have further implications in the gut microbiota, which is beyond the scope of this paper, but nevertheless warrants further research.

Serum lipids were largely affected by diets. A strong reduction effect was observed for total cholesterol, LDL-cholesterol, VLDL-cholesterol, total lipids and triacylglycerols by *L. digitata* inclusion. Even if total cholesterol slightly exceeded the reference values (36–54 mg/dL) for pigs^23^. Such beneficial hypocholesterolaemic effect is known to counterbalance cardiovascular risk factors^24^. In fact, the same finding has been reported for microalgae species, such as *Chlorella* sp.^25,26^. While *L. digitata* reduced glucose, increasing in parallel IGF-1, both insulin and insulin resistance index were unaffected by diets, suggesting the maintenance of glycaemia homeostasis. Insulin-like growth factor (IGF-1) has the ability to decrease blood glucose levels, which is in agreement with our data. Insulin is a well-known stimulator of lipogenesis^27^ and stimulates fatty acid synthesis in the liver with formation and storage of triacylglycerols^28^.

In general, the low levels and variations found for urea (average 6.65 mg/dL) and creatinine (average 0.654 mg/dL) indicate unaffected renal function. In this regard, the higher values reported by Jackson and Cockcroft^23^ (10–30 mg/dL and 1.0–2.7 mg/dL for urea and creatinine, respectively) and Coelho et al*.*^14^ are relative to adult pigs, whereas those shown in this study concern piglets, thus possibly justifying the discrepancy. Hepatic function markers, such as ALT, AST, GGT and ALP were all significantly decreased by *L. digitata* inclusion reaching the lowest activities without CAZymes. Despite the variations observed for aminotransferase activities, it is worth noticing that the levels found are close to reference values for pigs, which are 31–58 U/L for ALT, 32–84 U/L for AST, 10–52 U/L for GGT and 118–395 U/L for ALP, respectively^23^, which indicates that supplementation with *L. digitata* does not affect piglet liver health. Some algae have been reported to have immune-modulatory properties. For instance, their anti-inflammatory activity may be linked to a minor secretion of pro-inflammatory cytokines^29,30^. Our data indicate that *L. digitata* inclusion increased IgG and IgM immunoglobulins, which are the first line of defence of the organism against infections^31^. This ultimately contributed to an increased serum protein content. In particular, IgG and IgM antibodies act in a coordinated way in short- and long-term protection against infections^32^. Upon infection, the IgM level will rise for a short time and then will begin to drop as the IgG levels increase, protecting the organism in the long-term^32^. In accordance, white blood cells in general, particularly lymphocytes, were also increased in piglets fed *L. digitata*-based diets. Altogether, these changes suggest an improved immune response during the critical post-weaning period in piglets.

Sugiharto and Lauridsen^33^ and Barkia et al.^34^ proposed that omega-3 fatty acids are among the biological active compounds that confer immune-enhancing properties to algae. Abdelnour et al.^35^ also suggested that antioxidants, β-carotene and vitamin B12 available in seaweeds can modulate the immune function, even in different animal models such as broilers. IL-6, C-reactive protein and ApoA1 were found below the minimum detection limits while IL-10, a pleiotropic cytokine known for its potent anti-inflammatory and immunosuppressive effects that maintain normal tissue homeostasis^36^, was increased in piglets fed *L. digitata* diets, with or without feed enzymes supplementation. Taken together, these positive variations reflect a boost on cellular and humoral immune responses stimulated by *L. digitata* that assure piglets’ survival during the post-weaning period.

Cortisol is one of the most widely used biomarkers to detect stress in pigs^37^ and is the main glucocorticoid in this species^38^. Stress is a process with multifactorial causes, which produces an organic response that generates negative effects on animal health and welfare, and general productive performance. These include erratic behaviours such as tail biting, immune system suppression, among other effects that depend on the duration of the stress exposure^37^. Once again, *Laminaria*-based diets displayed a beneficial effect in piglets by promoting a reduction of this stress hormone indicating less metabolic stress, which often is the end-result of dietary changes in piglets^39^. In our experiment, the exogenous feed enzymes did not reverse such effect.

Among others, the most common electrolyte disorders associated with renal failure are Sodium and Potassium^40^. In addition, kidney plays a key role in maintaining chloride balance in the body. Renal Chloride transport is usually coupled to Sodium transport^41^, in accordance with the changes observed for both parameters in this study. The values found in this study for Sodium, Potassium and Chloride are within normal range for swine: 140–150, 4.7–7.1 and 95–103 mEq/L, respectively^23^, with the exception of Potassium in the Rovabio^®^ supplemented piglets. Although statistically significant, the variations found for the three electrolytes are devoid of clinical physiological relevance, as the mean values obtained are very similar among experimental diets coupled with a minimal standard error.

*Laminaria digitata* polysaccharides, fucose and glucans, have shown antioxidant activities by measuring ferric reducing antioxidant power (FRAP) and 1,1-diphenyl-2-picryl-hydrazyl (DPPH) radical scavenging activity^42^, although herein, the seaweed composition had no impact on systemic antioxidant defence as well as on the accumulation of hepatic antioxidant pigments. In a companion study by our research team, dietary seaweed has not resulted in the detection of muscle pigments in these piglets^16^.

The hepatic fatty acid profile was significantly influenced by experimental diets. Indeed, *L. digitata* diets significantly increased total n-3 PUFA in the liver, which explains the decreased n-6/n-3 ratio found also in this tissue. Major n-3 PUFA, such as C18:4n-3 and C20:5n-3 (EPA) were highly present in *L. digitata* diets, with the latter not being detected in the control group^16^. Therefore, dietary availability is most likely the major driver for such changes, similarly to what has been recently reported for the meat of these piglets^16^. EPA accumulation was significantly increased in *L. digitata* diets compared to control, regardless of enzyme supplementation. The same was reported in broiler chickens when fed with 15% dietary inclusion of this seaweed^43^, which contributes to improved nutritional composition of this edible tissue, in addition to increasing its health promoting properties for the piglet itself. Moreover, n-3 PUFA accumulation could contribute towards downregulation of PUFA oxidation pathways and improved hepatic oxidative status^44^. Feeding 2% of brown seaweed lipids to mice has also significantly increased hepatic n-3 PUFA content, including 22:6n-3 that is the end-product of 18:3n-3 elongation pathway^45^. In this study, there was a significant hepatic increase of the latter FA in *L. digitata* diets accompanied by a strong tendency to increase 22:6n-3. Similar to what has been reported by Coelho et al.^14^ using 5% of dietary *Chlorella vulgaris* in finishing pigs, increased hepatic n-3 PUFA contents could be at least partially explained by the increased intake of the major precursor of PUFA elongation and desaturation, 18:3n-3 fatty acid. Contrary to what was recorded in some of the serum parameters, the effect of seaweed was again not reversed by enzymatic supplementation.

Seaweeds in general have high ash/mineral contents, which may have beneficial or detrimental effects depending on factors such as species and dietary inclusion levels^2,4^. Indeed, a recent study has found that feeding pigs with up to 4% *Macrocystis pyrifera* increases total ash in pigs’ meat^46^. Herein, we found that Phosphorus and Sulphur were significantly reduced in the liver of piglets fed *L. digitata* supplemented with Rovabio^®^ and the recombinant alginate lyase compared to control and *L. digitata* groups, which explains the concomitant reduction found in total macrominerals content. An earlier study has reported that 15% of dietary *L. digitata* actually has the reverse tendency on the hepatic mineral content of broilers^43^. In this study, Phosphorous levels were lower in seaweed diets, which could explain such differences. However, it was not the case of Sulphur, which was at least 47% higher in seaweed diets. Several authors have previously reported that seaweeds are a good source of microelements, but such an effect on hepatic Sulphur content has not been reported^47^. Accordingly, Copper and Manganese were significantly reduced in *L. digitata* with or without the recombinant alginate lyase compared to control, and also in *L. digitata* with the recombinant alginate lyase compared to *L. digitata* alone. These microelements are particularly important as cofactors for antioxidant enzymes, such as the superoxide dismutase^48,49^. The lower presence of these minerals in *L. digitata*-fed piglets could indicate a lower SOD activity, since they also accumulated n-3 PUFA which have a down-regulating effect on reactive-oxygen species generating processes, namely FA oxidation. In our study, we did not evaluate hepatic oxidative parameters, thus this putative mechanism requires additional research.

## Conclusions

The findings indicate a strong impact of seaweed on blood cells, serum lipids, immunoglobulins, IL-10 and cortisol, thus enhancing the health response of piglets during the weaning period. *L. digitata* improved the cellular and humoral immune system by increasing lymphocytes and IgG and IgM levels. Additionally, the seaweed had hematopoietic, anti-inflammatory and hypolipidemic effects, and positively influenced piglet wellbeing as indicated by lower cortisol levels. Furthermore, the liver showed increased levels of n-3 PUFA and upregulation of fatty acid elongation pathways. The inclusion of seaweed did not have any adverse effect when combined with feed enzymes. The reduction in micro and macromineral levels in pigs fed with seaweed diets suggests a differential mineral bioavailability.

Further research is nevertheless necessary to fully understand the underlying hematopoietic, immunostimulatory, anti-inflammatory and hypolipidemic mechanisms, and to evaluate the impact of dietary *L. digitata* on mineral bioavailability. A complementary approach based for instance on proteomics and metabolomics could also provide valuable insights into how the hepatic metabolism is affected by the feed incorporation of seaweed and feed enzyme supplementation.

## Materials and methods

### Animal trial and experimental design

The principles and guidelines of the European Union legislation (2010/63/EU Directive), as well as the ARRIVE guidelines 2.0 (https://arriveguidelines.org/arrive-guidelines), procedures were used in this animal experiment. It was reviewed by the Animal Experimentation Ethics Commission of the Higher Institute of Agronomy of the University of Lisbon (Portugal). It was authorized by the Animal Care Committee of the National Veterinary Authority (Process Number 0421/000/000/2020, *Direção Geral de Alimentação e Veterinária*, Lisbon, Portugal). The experimental design and diet composition have been thoroughly described in detail in a companion paper^16^. Control and *L. digitata* diets had 18% and 17% crude protein on a dry matter (DM) basis and crude fat averaged 4.15% on a DM basis. The seaweed had 4.85% and 1.31% crude protein and crude fat on a DM basis, respectively. For further details, please refer to our companion paper^16^. Briefly, the animal trial was conducted at the Animal Production Section of the Higher Institute of Agronomy, University of Lisbon, Portugal. We randomly allocated 40 recently weaned male piglets (Large White × Duroc) into four experimental diets (n = 10): a control diet based on wheat, maize and soybean meal, LA (10% *L. digitata*, replacing control), LAR (LA + 0.005% Rovabio^®^ Excel AP of Adisseo (Antony, France) and LAL (LA + 0.01% alginate lyase). After an adaptation period of 5 days to experimental conditions, the trial started and lasted for two weeks, after which all piglets were slaughtered following standard commercial practices. Liver samples were collected, minced and frozen at – 20 °C. Blood was collected and centrifuged at 1500*g* for 15 min for serum collection, which was frozen at – 80 °C, until further analysis.

### Blood profile measurements

Serum biochemical profile analysis has been previously described by Madeira et al.^15^. Triacylglycerols (TAG), total cholesterol, phospholipids, urea, total protein, LDL-cholesterol, HDL-cholesterol, glucose and creatinine concentrations and serum hepatic markers were determined in a Modular Hitachi Analytical System (Roche Diagnostics, Mannheim, Germany) using diagnostic kits (Roche Diagnostics, Meylan, France) following manufacturer’s instructions. Covaci et al.^50^ and Friedewald et al.^51^ formulas were used to calculate VLDL-cholesterol and total lipids. Immunoglobin profile (IgA, IgG and IgM) was determined by immunoturbidimetry. Quanti-ChromTM Antioxidant Assay Kit (DTAC-100, Bioassay Systems, Hayward, CA, USA) was used for the determination of total antioxidant capacity and EnzyChromTM Glutathione Peroxidase Assay Kit (EGPx-100, Bioassay Systems, Hayward, CA, USA) for glutathione peroxidase activity (GPx). One unit of GPx is the amount of GPx that produces 1 μmol of glutathione disulphide (GS-SG) per minute at pH = 7.6 and room temperature. Blood counts (white blood cells, thrombocytes and red blood cells) were performed using Sysmex XN-10 analysers (Sysmex Corporation, Kobe, Japan), as reported^14^. Insulin growth factor-1 (IGF1), interleukin-6 (IL-6) and cortisol were determined with an electrochemiluminescence immunoassay kit (Roche Diagnostics, Meylan, France), as described^52^. Apolipoprotein A1 (ApoA1) and C-reactive protein were determined by immunoturbidimetry (Roche Diagnostics, Meylan, France). IL-10 was determined with an immunoassay kit supplied by DIASource (Louvain-la-Neuve, Belgium).

### Hepatic lipid profiling

Freeze-dried liver samples were used for total lipid extraction, using the Folch et al.^53^ method with methanol and dichloromethane (1:2 v/v)^54^. Fatty acids were transesterified using NaOH in anhydrous methanol (0.5 M) followed by methanol:HCl (1:1 v/v) at 50 °C, for 30 and 10 min, respectively^55^. Fatty acid methyl esters were determined with gas-chromatography (Hewlett-Packard, Palo Alto, CA, USA) with a flame-ionization detector and a CP-Sil 88 capillary column (100 m, 0.25 mm, 0.20 µm film thickness; Chrompack, Varian Inc., Walnut Creek, CA, USA), as previously reported^56^. Nonadecanoic acid (19:0) was selected as internal standard, converting peak areas into weight percentages. Fatty acids were identified according to retention times and presented as g/100 g of total fatty acids.

### Liver pigments analysis

Chlorophylls a, b and total carotenoids were determined using the procedure described by Teimouri et al.^57^. Pigments were determined by adding 10 mL of acetone to 1 g of fresh tissue, followed by incubation at room temperature with agitation overnight. Samples were then centrifuged at 1500*g* for 5 min and measured using UV–Vis spectrophotometer (Amersham Biosciences, Little Chalfont, UK). The pigment content was calculated using the equations described by Hynstova et al.^58^.

### Liver mineral profile

Hepatic mineral profile was determined according to Ribeiro et al.^59^. Briefly, 0.3 g of freeze-dried tissue were weighed into a digestion tube. Concentrated solutions of HCl and HNO_3_ were added to each tube, followed by an overnight incubation. Before digestion, H_2_O_2_ was added to each tube followed by 1 h of gradual increase to 95 °C and another hour at constant 95 °C. The resulting solution was then filtered and analysed using Inductively Coupled Plasma—Optical Emission Spectrometry (ICP-OES).

### Statistical analysis

Data was analysed using the General Linear Model (GLM) procedure of the SAS software (version 9.4, SAS Institute Inc., Cary, NC, USA), using the piglet as the experimental unit. Statistically significant differences were compared with the Tukey test of the PDIFF option. Standard errors of the means (SEM) were obtained using the univariate procedure. The significance level was set at P < 0.05. Blood and liver data were further analysed by a Principal Component Analysis (PCA) with SPSS Statistics for Windows (IBM Corp. released 2017, version 25.0, Armonk, NY, USA).

## Data Availability

All data is contained in the article.
